# Causes and Clinical Outcomes of Acute Kidney Injury After Cardiac Arrest: A Retrospective Cohort Study

**DOI:** 10.3390/medicina61020338

**Published:** 2025-02-14

**Authors:** Murat Aslan, Rabia Yılmaz, Dicle Birtane, Zafer Çukurova

**Affiliations:** 1 Anesthesia and Reanimation Clinic, Bakırköy Dr Sadi Konuk Training and Research Hospital, University of Health Sciences, Istanbul 34140, Türkiye; drrabiayilmaz@gmail.com (R.Y.); dicle1tane@gmail.com (D.B.); zcukurova@gmail.com (Z.Ç.); 2 Department of Anesthesiology and Reanimation, Gaziantep City Training and Research Hospital, Gaziantep 27470, Türkiye

**Keywords:** cardiac arrest, acute kidney injury, body mass index

## Abstract

*Background and Objectives:* The development of acute kidney injury (AKI) in the post-cardiopulmonary resuscitation (post-CPR) period is a common pathology that has not been adequately investigated but contributes significantly to morbidity and mortality. We aimed to investigate the causes of AKI in the early post-CPR period. *Materials and Methods:* This study was performed retrospectively in 82 adult patients who survived for at least 2 days out of 312 patients admitted to the intensive care unit after cardiac arrest in 2013–2022. AKI developed in 40 (48.7%) of these 82 patients (AKI 1–3 patient, respectively: 14, 13, 13). Binary logistic regression analysis was performed separately to determine the risk factors for AKI and mortality. *Results:* Each unit increase in BMI increased the risk of developing AKI by 1.272-fold, and the increase was statistically significant [OR (95%CI) = 1.272 (1.089–1486); *p* = 0.002]. The use of VSP and INO treatment alone increased the risk of AKI by approximately 14-fold, and this increase was statistically significant [OR (95%CI) = 14.225 (1.172–172.669); *p* = 0.037]. The combined use of VSP and INO treatment increased the risk of AKI by approximately 42-fold, and this increase was statistically significant [OR (95%CI) = 42.089 (2.683–660.201); *p* = 0.008]. The COVID-19 period alone increased the risk of developing AKI by 2.8-fold compared to the non-COVID-19 period, but the statistical significance of this increase was limited [OR (95%CI) = 2.801 (0.859–9.126); *p* = 0.088]. The development of AKI was not associated with mortality [OR (95%CI) = 2.194 (0.700–6.872); *p* = 0.178]. *Conclusions:* Having VSP and/or INO support and high BMI in the post-CPR period are the most important reasons for the development of AKI. COVID-19 may also increase the risk of developing AKI.

## 1. Introduction

Cardiac arrest refers to the cessation of mechanical activity of the heart. It can occur in or out of hospital. The incidence of in-hospital cardiac arrest occurs in 1–1.5 out of every 1000 hospital admissions [1]. Out-of-hospital cardiac arrest can occur in 50–120 people per 100,000 [1,2]. Return of spontaneous circulation (ROSC) is associated with time of CPR initiation, quality of CPR, and many demographic variables [1,2]. Also, in recent years, medical emergency research has focused on the secondary impact of COVID-19 and the modification of the emergency medical system [2].

In the post-cardiopulmonary resuscitation (post-CPR) period, if ROSC can be achieved, intensive care unit (ICU) follow-up is required for treatment management. In the post-CPR period, in addition to treating the cause of cardiac arrest, ischemia and reperfusion damage to the brain, kidneys, and other organs should be minimized. Depending on the duration of cardiac arrest and the adequacy of post-CPR care, permanent neurological, renal, and other organ damage may develop [3].

After the brain, heart, and kidneys are the most susceptible organ to ischemic damage [4]. Prolonged return of ROSC, non-shockable initial rhythm, and post-resuscitation shock are the most recognized risk factors for AKI [5]. If the duration of ischemia is shorter than 30 min, permanent renal damage is not expected [6]. Even if the duration of CPR does not always exceed 30 min, renal damage may develop due to ongoing hypotension and hypoxemia. In addition, age, gender, body mass index, comorbidities, and COVID-19 may increase the risk of developing AKI. Therefore, post-resuscitation care is also very important in terms of the prevention of organ damage. Hypoxic–ischemic brain injury accounts for approximately two-thirds of in-hospital deaths in comatose patients admitted to the ICU after out-of-hospital cardiac arrest [7]. However, the development of AKI after cardiac arrest is also a common complication that severely affects the prognosis [5,8].

It is important to identify the causes that increase the risk of AKI development in order to optimize treatment and reduce mortality after cardiac arrest. The aim of this study was to investigate the reasons that increase the risk of early AKI in patients admitted to the ICU in the post-CPR period.

## 2. Materials and Methods

### 2.1. Study Design and Patient Population

This study was conducted retrospectively on patients admitted to the Anesthesiology and Reanimation ICU of Bakırköy Dr Sadi Konuk Training and Research Hospital between 2013 and 2022 after a witnessed out-of-hospital cardiac arrest or in-hospital cardiac arrest.

**Figure 1:**
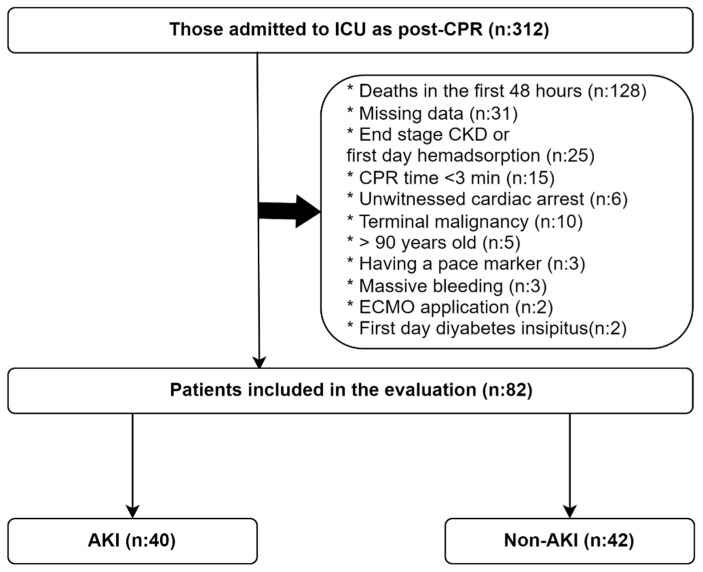
Flowchart of the study.

Patients included in this study were identified according to the following flow chart. The total number of patients admitted to the ICU after cardiac arrest was 312. According to the current KDIGO 2012 AKI criteria, creatinine must be monitored for at least 48 h to diagnose AKI, apart from urine output (≥0.3 mg/dL increase is required). Therefore, 128 patients who died before 48 h were excluded. We excluded 31 patients with missing data on CPR or other parameters. Twenty-five patients with end-stage renal failure or using hemadsorption techniques that would complicate the diagnosis of AKI were excluded from the study. We excluded 15 patients with very short CPR time (<3 min) because the risk of impaired renal perfusion and AKI development was very low. We excluded 6 patients with unwitnessed arrest because the duration of renal perfusion deterioration was unknown. In addition, patients with terminal malignancy (*n*: 10), pace-maker (*n*: 3), massive bleeding (*n*: 3), ECMO (*n*: 2), early diabetes insipidus (*n*: 2), and age > 90 years (*n*: 5) were excluded, as these may lead to the development of AKI and complicate its diagnosis. Of these patients, 82 were eligible for inclusion in this study (Figure 1).

**Figure 2:**
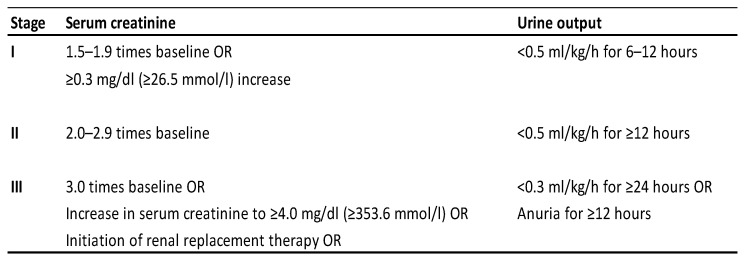
KDIGO 2012 clinical practice guidelines for acute kidney injury (AKI) [9].

Patients were divided into AKI (*n*: 40) and non-AKI (*n*: 42) groups according to urine output and creatinine level increase using KDIGO 2012 AKI criteria [9]. Serum basal creatinine levels measured immediately after or shortly before cardiac arrest (within 3 months). According to the KDIGO 2012 criteria, an increase of ≥0.3 mL in the basal creatinine level within 48 h, a ≥1.5-fold increase within seven days, or a urine output of <0.5 mL/kg/h for 6 h are considered AKI. Of the 40 patients who developed AKI, 14 were evaluated in the AKI-1 group, 13 in the AKI-2 group, and 13 in the AKI-3 group (Figure 2).

### 2.2. Data Collection

Patient data registered in ImdSoft-Metavision/QlinICU Clinical Decision Support Software in the ICU of the Anesthesia and Reanimation Clinic were obtained through structured query language (SQL) queries.

Demographic data (age, gender, weight, height), heart rate, mean arterial pressure, pupillary reflex, GCS (Glasgow coma scale), site of cardiac arrest, duration of CPR, comorbidities, arterial blood gas, PaO_2_/FiO_2_ ratio, hemogram, glucose, creatinine, aspartate aminotransferase (AST), alanine aminotransferase (ALT), Na, CI, K, C-reactive protein (CRP), renal replacement therapy (RRT), targeted temperature management (TTM), vasopressor/inotrope requirement, invasive interventional procedures, fluid balance, ICU length of stay, and survival status were obtained from the decision support system. The initial cardiac rhythms (non-shockable/shockable) of the patients after CPR could not be evaluated due to insufficient data.

The weight of the patients is obtained by measurement from beds with scales. Height values are also measured in the supine position. BMI values were calculated using the formula weight (kg)/height (meters)^2^. The first 24 h heart rate (HR) and mean arterial pressure (MAP) values of all patients were analyzed and the mean value was calculated. CCI (Charlson comorbidity index) and APACHE-II score were calculated using the data in the decision support system (The Charlson comorbidity indexes of the patients. This was calculated by entering patient data from the https://www.mdcalc.com/calc/3917/charlson-comorbidity-index-cci (accessed on 1 January 2024) website; APACHE-II scores of the patients. This was calculated by entering patient data from the https://www.mdcalc.com/calc/1868/apache-ii-score (accessed on 1 January 2024) website. The AKI score of the patients was calculated using 2012 KDIGO criteria (Figure 2).

### 2.3. Treatment Protocol

CPR is performed according to the current European Resuscitation Council guidelines in all patients admitted to our hospital with out-of-hospital or in-hospital cardiac arrest. In patients with the return of spontaneous circulation, respiratory safety is usually ensured by orotracheal intubation. During or after CPR, necessary laboratory tests and body imaging methods (echocardiography, electrocardiography, computed tomography, magnetic resonance imaging) are performed to investigate cardiac, respiratory, neurological, and other etiological causes. These patients are then assessed for their requirement of coronary angiography, cerebral angiography, or other interventional procedures (tube thoracostomy, decompressive craniectomy, laparotomy, etc.).

Patients whose spontaneous circulation is restored are usually admitted to the ICU within 2–3 h after the necessary laboratory, imaging, and interventional (coronary or cerebral angiography) procedures for the cause and treatment are performed.

Post-cardiac arrest treatment management was performed in accordance with ESICIM guidelines.

### 2.4. Statistical Analysis

Statistical analysis was performed using IBM SPSS Statistics for Windows, version 25.0 (IBM Corp., Armonk, NY, USA). The Kolmogorov–Smirnov test was used to determine whether the data were normally distributed. Descriptive statistics were given as a frequency (*n*) and a percentage (%) for the categorical variables and median + interquartile range (IQR) for the numerical variables. The Mann–Whitney U test was used to evaluate the statistical difference between quantitative variables between both groups. The Chi-squared test (Pearson Chi-square, continuity correction, or Fisher’s exact test) was used to compare categorical variables.

Binary logistic regression analysis was performed separately to determine the factors associated with the risk of AKI and mortality. First, univariable binary logistic regression analysis was performed. Multivariable regression analysis was then performed with the parameters that were considered clinically relevant, had a *p*-value < 0.2, and generated the highest Nagelkerke R-squared value. Statistical significance was accepted as a *p*-value < 0.05.

## 3. Results

### 3.1. Demographics and Clinical and Treatment Data

There was no statistically significant difference between the AKI and non-AKI groups in terms of the location of the witnessed cardiac arrest (in-hospital/out-of-hospital) (*p* = 0.518). In both patient groups, the majority of patients admitted to the ICU were similarly admitted from the emergency department (the rest were admitted from inpatient clinics or the operating room) (*p* = 0.951). Acute coronary syndrome (ACS) was the most common cause of cardiac arrest in both patient groups (*p* = 1.000). Respiratory problems were the second most common cause of cardiac arrest in both patient groups (0.947). The majority of patients who developed AKI were COVID-19 patients, and this rate was statistically higher than in the non-AKI group [respectively, 24 (60%) and 15 (35.7); *p* = 0.048]. There was no statistically significant difference in CPR times between the AKI and non-AKI groups [15 (10–22); 15 (10–28); *p* = 0.877] (Table 1).

There was no statistically significant difference between the AKI and non-AKI groups in terms of age, gender, and Charlson comorbidity index (CCI) data (*p* > 0.05). Body mass index (BMI) values were statistically higher in the AKI group [27.4 (24.7–34.0); 24.2 (22.9–26.7); *p* = 0.001]. There was no statistical difference between both groups in terms of mean HR and MAP values on the first day (*p* = 0.199; *p* = 0.285). APACHE-II mortality score values were statistically much higher in the AKI group [28 (19–32); 21 (17–26); *p* = 0.003]. There was no statistically significant difference in admission PaO_2_/FiO_2_ ratio values between AKI and non-AKI groups (*p* = 0.095). The number of patients with a GCS score < 4 in the neurologic evaluation performed on day 3–4 was statistically significantly higher in the AKI group [22 (55.0); 10 (23.8); *p* < 0.008]. There was no statistically significant difference in the rate of pupillary reflex negativity on day 3–4 in the AKI and non-AKI groups (*p* = 0.735). There was no statistically significant difference in baseline serum creatinine levels in both groups (*p* = 0.450). Serum creatinine values 2 days after cardiac arrest were statistically much higher in the AKI group [1.8 (1.2–3.0); 0.8 (0.6–1.0); *p* < 0.001]. In the AKI group, the serum pH and HCO_3_- levels were statistically lower (*p* = 0.015; *p* = 0.042), while the lactate and *p* (phosphorus) values were higher (*p* = 0.025; *p* = 0.005) than the ICU admission laboratory values. There was no statistical difference between the other laboratory admission parameters (*p* > 0.05) (Table 2).

There was no statistically significant difference in 24 h fluid input in the AKI and non-AKI groups [3.0 (1.7–4.4); 3.0 (2.5–4.0); *p* = 0.564]. Urine output in the first 24 h was statistically higher in the AKI group [1.0 (0.4–2.8); 1.9 (1.4–2.7); *p* = 0.001]. Fluid balance in the first 24 h was statistically higher in the AKI group [1.5 (0.9–3.1); 0.9 (0.0–2.1); *p* = 0.033]. The need for VSP (vasopressor) and/or INO (inotrope) in the first 24 h was significantly higher in the AKI group (*p* = 0.001). There was no statistically significant difference between both groups in terms of max body temperature on the first day (*p* = 0.314). There was no statistically significant difference in the number of patients who underwent targeted temperature management (TTM) in the first 24 h in both groups (*p* = 0.265). There was no difference in the number of patients requiring continuous RRT during ICU follow-up in both patient groups (*p* > 0.686). ICU length of stay was statistically shorter in the AKI group [5.6 (3.2–9.7); 9.3 (5.7–16.0); *p* = 0.020]. ICU mortality rate was statistically much higher in the AKI group [26 (65.0); 11 (26.2); *p* = 0.001] (Table 3).

### 3.2. Logistics Regression Analysis for AKI

Univariable and multivariable binary logistic regression analysis was performed to determine the factors affecting the risk of AKI development. Univariable analysis: age, gender, and CCI parameters were not associated with the risk of developing AKI (*p* > 0.05). Each unit increase in BMI was associated with a 1.153-fold increase in the risk of developing AKI, and this association was statistically significant [OR (95%CI) = 1.153 (1.038–1.280); *p* = 0.008]. No statistical relationship was found between the duration of CPR and the risk of AKI development (*p* = 0.946). There was no difference in the risk of AKI in cardiac arrest due to ACS compared to cardiac arrest due to other causes (*p* = 0.829). The COVID-19 period increased the risk of AKI development by 2.7-fold compared to the non-COVID-19 period, and this increase was statistically significant [OR (95%CI) = 2.700 (1.105–6.599); *p* = 0.029]. There was no statistically significant difference in AKI risk between out-of-hospital witnessed cardiac arrest and in-hospital witnessed cardiac arrest (*p* = 0.368). Mean HR and MAP parameters in the first 24 h were not statistically significantly associated with the risk of AKI development (*p* = 0.210; *p* = 0.291). The need for VSP or INO treatment alone increased the risk of developing AKI by 13-fold compared to no need for VSP and INO, and this association was statistically significant [OR (95%CI) = 13.000 (1.574–107.354); *p* = 0.017]. The need for VSP and INO increased the risk of AKI by 39-fold compared to no VSP and INO, and this association was highly statistically significant [OR (95%CI) = 39.000 (4.022–378.199); *p* = 0.002]. TTM treatment was not statistically associated with the development of AKI (*p* = 0.165). Each unit increase in admission phosphorus value was associated with a 1.367-fold increase in the risk of AKI development, and this relationship was statistically significant [OR (95%CI) = 1.367 (1.086–1.719); *p* = 0.008]. With each unit of pH increase, the probability of developing AKI decreases by 1/0.021 = 47.619 times and vice versa (with each unit of pH decrease, the probability of developing AKI increases 47.619 times). This would mean that a 0.01 (1/100 = 0.01) unit decrease in admission pH is associated with a 47.619% (47.619/100 = 0.47619 = 47.619%) increased probability of developing AKI [OR (95%CI) = 0.021 (0.001–0.641); *p* = 0.027]. Each unit increase in admission lactate value was associated with a 1.263-fold increase in the risk of AKI development, and this relationship was statistically significant [OR (95%CI) = 1.263 (1.045–1.526); *p* = 0.016]. Admission creatinine, WBC, hemoglobin, and glucose parameters were not associated with the risk of AKI development (*p* > 0.05). There was no statistical relationship between the change in the admission PaO_2_/FiO_2_ ratio and the risk of developing AKI (*p* = 0.223). There was no statistical relationship between the amount of fluid intake in the first 24 h and the risk of AKI development (*p* = 0.950) (Table 4).

Multivariable analysis: A model was created by examining the relationship between BMI, COVID-19 period, VSP and/or INO need, admission lactate, and admission phosphorus parameters with AKI. Each unit increase in BMI increased the risk of developing AKI by 1.272-fold, and the increase was statistically significant [OR (95%CI) = 1.272 (1.089–1486); *p* = 0.002]. The COVID-19 period alone increased the risk of developing AKI 2.8-fold compared to the non-COVID-19 period, but the statistical significance of this increase was limited [OR (95%CI) = 2.801 (0.859–9.126); *p* = 0.088]. The use of VSP and INO treatment alone increased the risk of AKI by approximately 14-fold, and this increase was statistically significant [OR (95%CI) = 14.225 (1.172–172.669); *p* = 0.037]. The combined use of VSP and INO treatment increased the risk of AKI by approximately 42-fold, and this increase was statistically significant [OR (95%CI) = 42.089 (2.683–660.201); *p* = 0.008]. There was no statistical relationship between admission lactate and phosphorus parameters and AKI development (*p* = 0.230; *p* = 0.113) (Table 4).

### 3.3. Logistics Regression Analysis for Mortality

Univariable and multivariable binary logistic regression analysis was performed to determine the factors affecting the risk of mortality development. In univariable analysis, there was no statistically significant relationship between age, gender, CCI, BMI, and the duration of CPR and the risk of mortality (*p* > 0.05). There was no statistically significant difference in mortality risk in cardiac arrest due to ACS compared to cardiac arrest due to other causes (*p* = 0.178). There was no statistically significant difference in mortality risk in out-of-hospital witnessed cardiac arrest compared to in-hospital witnessed cardiac arrest (*p* = 298). Each unit increase in mean HR in the first 24 h was associated with a 1.032-fold increase in the risk of mortality, and this association was statistically significant [OR (95%CI) = 1.032 (1007–1058); *p* = 0.010]. There was no statistical relationship between a change in the mean MAP value in the first 24 h and mortality risk (*p* = 0.351). The use of VSP or INO alone was not significantly associated with mortality compared to no VSP or INO [OR (95%CI) = 4.667 (0.940–23.158); *p* = 0.059]. Both VSP and INO use were associated with a 14-fold increase in mortality compared to no VSP and INO use, and this association was highly statistically significant [OR (95%CI) = 14.000 (2.370–82.717); *p* = 0.004]. The development of AKI 1–3 was associated with a 5.2-fold increase in mortality risk and this association was statistically significant [OR (95%CI) = 5.234 (2.032–13.482); *p* = 0.001]. A GCS score < 4 was associated with a 5.133-fold increase in mortality risk, and this association was statistically significant [OR (95%CI) = 5.133 (1.962–13.428); *p* = 0.001]. A negative pupillary reflex was associated with a 12.138-fold increase in mortality risk, and this association was statistically significant [OR (95%CI) = 12.138 (1.441–102.247); *p* = 0.022]. Each unit increase in admission lactate value was associated with a 1.239-fold increase in mortality risk, and this relationship was statistically significant [OR (95%CI) = 1.239 (1.032–1.488); *p* = 0.022]. Each unit increase in the admission PaO_2_/FiO_2_ ratio was associated with a 1.005-fold decrease in mortality risk, and this relationship was statistically significant [OR (95%CI) = 0.995 (0.992–0.999); *p* = 0.023]. There was no statistically significant association between the risk of mortality and change in admission WBC, hemoglobin, and glucose values (*p* > 0.05). There was no statistically significant relationship between fluid intake in the first 24 h and the risk of mortality [OR (95%CI) = 1.241 (0.965–1.594); *p* = 0.092]. There was no statistically significant association between the need for continuous RRT and mortality risk (*p* > 0.115) (Table 5).

Multivariable analysis: A model was created by examining the association of the need for VSP and/or INO, the development of AKI, GCS < 4, and the PaO_2_/FiO_2_ ratio with mortality. There was no statistical association between the need for VSP or INO and mortality risk [OR (95%CI) = 2.863 (0.497–16.495); *p* = 0.239]. The need for VSP and INO increased the mortality risk 8.391-fold compared to no need for VSP and INO, and this association was statistically significant [OR (95%CI) = 8.455 (1.132–63.160); *p* = 0.037]. The development of AKI 1–3 alone was not associated with an increased risk of mortality [OR (95%CI) = 2.194 (0.700–6.872); *p* = 0.178]. A GCS value of <4 alone increased the risk of mortality by 4.529 times, and this relationship was statistically significant. [OR (95%CI) = 5.004 (1.590–15.750); *p* = 0.006]. Each unit increase in the PaO_2_/FiO_2_ ratio alone decreased the risk of mortality by 1.005-fold, and this decrease was statistically significant [OR (95%CI) = 0.995 (0.991–1.000); *p* = 0.044] (Table 5).

## 4. Discussion

The primary aim of this study was to determine the reasons that increase the risk of AKI development in the early period after cardiac arrest. AKI was detected in 40 (48.8%) of 82 patients admitted to an intensive care unit (ICU) after cardiac arrest. However, since the incidence of AKI only includes patients who survive more than 48 h after cardiac arrest, this rate may be higher in reality. Current studies on this subject also show an incidence of AKI of around 50% after cardiac arrest [10,11]. As a result of our study, we found that high BMI and the need for VSP and/or INO were both independent risk factors for the development of AKI. Recent studies have also shown that a high BMI index is associated with an increased risk of AKI in critically ill patients [12,13,14,15]. Although the exact mechanism of the increased frequency of AKI in patients with increased BMI is unknown, altered renal hemodynamics, increased leptin production, decreased adiponectin production, high HDL levels, impaired autophagy system, impaired renal perfusion due to increased intra-abdominal pressure, other comorbidities, metabolic syndrome, and difficulty in intravascular volume assessment have been blamed [12].

Although the duration of CPR alone may be a sufficient factor for the development of AKI, the need for VSP and/or INO and high blood lactate levels in the first 24 h in our study suggest that AKI patients have more severe ongoing tissue perfusion impairment. Post-cardiac arrest syndrome has many similar features to sepsis, including decreased intravascular volume, vasoplegia, endothelial damage, and microcirculatory abnormalities [16,17,18]. Significant myocardial dysfunction is common after cardiac arrest, typically beginning to improve in 2–3 days, although full recovery takes significantly longer [3,19]. Whole-body ischemia/reperfusion injury after cardiac arrest, CPR, and return of spontaneous circulation also contribute to multiple organ failure [3].

In our study, unlike the non-AKI group, most of the group that developed AKI consisted of patient groups admitted during the COVID-19 pandemic. In addition, although the statistical significance was limited, it was determined that the development of cardiac arrest during the COVID-19 period was associated with an increased risk of AKI. Recent studies report an increase in cardiac arrest and deaths due to non-ST myocardial infarction during the COVID-19 pandemic due to the stay-at-home policy and the delayed responses of emergency medical systems [20,21]. Although COVID-19 primarily affects the lungs, the risk of AKI development due to kidney involvement is also high. Although the exact mechanism is still unclear, studies have found that AKI develops in >20% of patients admitted to hospital and >50% of patients admitted to the ICU [22,23].

Secondarily, we also investigated the effect of AKI development on mortality. The ICU admission Acute Physiology and Chronic Health Evaluation (APACHE-II) mortality assessment score was higher in patients with AKI compared to the non-AKI group. Since creatinine value is also a parameter of the APACHE-II score, it was expected that mortality would be higher in the AKI group. The mortality rate in patients who developed AKI was approximately 2.5 times that of the non-AKI group (65%). Recent studies on this subject show that the mortality rate in patients with AKI is over 60% [10,11]. In our study, AKI development was associated with increased mortality, but AKI development was not an independent risk factor for increased mortality. In our study, the need for VSP and INO, a low PaO_2_/FIO_2_ ratio, and severe neurologic damage (GCS motor score < 4) in the post-cardiac arrest period were independent risk factors for mortality. Providing hemodynamic stabilization in the post-cardiac arrest period is the most important common factor in terms of both the prevention of renal damage and the reduction of mortality. In our study, the most probable reason for the shorter duration of ICU hospitalization in patients with AKI may be due to higher early mortality.

In our study, it was not expected that there would be no statistical relationship between the time/place of CPR application (in-hospital and out-of-hospital) and the development of AKI and mortality. AKI is one of the components of post-cardiac arrest syndrome. In the post-resuscitation care section of the ERC 2021 Resuscitation guideline, it is stated that the severity of post-cardiac arrest syndrome is associated with the duration of CPR [3]. In a review conducted by Lars W. Andersen et al. in 2019, it was found that the mortality rate in in-hospital cardiac arrest patients was higher than in out-of-hospital cardiac arrest patients [24]. The main reason for the lack of a statistical relationship between these risk factors and AKI may be that patients with exitus within the first 2 days were excluded from the study, and the overall CPR times in these patients were not long enough to cause AKI. In humans, permanent kidney damage is not expected to occur until the duration of ischemia exceeds 30 min [6].

There was no association between the cause of cardiac arrest (ACS and non-ACS) and the development of AKI in our patients. In patients with cardiac arrest of primary cardiac origin, the initial rhythm is a more frequently shockable rhythm compared to arrests of a non-cardiac origin [25]. A non-shockable first rhythm during resuscitation was associated with a higher risk of AKI [10]. However, these data could not be obtained in our study. Patients with cardiac arrest due to ACS were admitted after routine coronary angiography. Contrast nephropathy may also contribute to the development of AKI in this patient group [26,27]. However, there was no increased risk of AKI development and mortality in this patient group.

In our study, we could not obtain any data directly linking the necessity of CRRT to the development of cardiac arrest. There may be many factors (sepsis, hypovolemia, toxic causes, etc.) that lead to acute kidney failure requiring continuous RRT other than cardiac arrest during ICU follow-up. Therefore, it is difficult to evaluate this as a direct consequence of cardiac arrest. We also did not find any association of CRRT with mortality in this patient group. However, this may be due to the fact that although the proportion of patients who developed AKI was close to 50%, the number of patients who underwent CRRT was very low (14.6%). This could be the theory that RRT is not tolerated due to hemodynamic instability or low life expectancy.

In our study, the amount of fluid intake in the first 24 h was similar in both groups, and no association was found with the development of AKI. Although mortality was higher in those with higher fluid intake in the first 24 h, the association with mortality was not sufficiently statistically significant. The main indication for fluid replacement in intensive care patients is hemodynamic stabilization and prevention of AKI [28]. We use invasive (pulse contour analysis, transpulmonary thermodilution) and non-invasive (echocardiography, pleth variability index) hemodynamic monitoring methods to determine the amount of fluid replacement in critical intensive care patients. We also apply a restrictive fluid management strategy in patient treatment management. In a retrospective cohort study conducted by Christoph Adler et al. in 2012, it was found that the incidence of AKI development was statistically reduced in the group of patients monitored with PICCO and given higher fluid volume in the first 24 h (4375 ± 1285 mL vs. 5449 ± 1438 mL; *p* = 0.007) [8]. However, in recent studies, there is increasing evidence that hypervolemia increases the incidence of AKI in critically ill intensive care patients by causing renal congestion and impairing intra-abdominal perfusion [28]. Therefore, a targeted controlled fluid management strategy using invasive or non-invasive hemodynamic monitoring methods may be more useful in this patient group.

Our most important limitations were that this study was single-centered and retrospective. It is very difficult to conduct a single-center prospective control study in this patient group because of the multiplicity of causes leading to cardiac arrest, high early mortality, and a wide variety of factors that may lead to AKI. Data on who administered CPR in out-of-hospital cardiac arrest cases (bystander or emergency medical services team) were missing and could not be analyzed. This may have an impact on the quality of CPR, the degree of hypoperfusion, and, consequently, the risk of AKI. However, since the rate of out-of-hospital cardiac arrest was relatively lower in the group with AKI, such a significant effect was not considered. The lack of data on the first cardiac arrest rhythm, which is directly related to AKI development and mortality, was an important limitation. It is difficult to determine long-term outcomes in patients who develop AKI because neurologic damage is the most commonly affected and the leading cause of mortality in post-CPR patients. The fact that our study data were obtained from an electronic decision support system was important for data security.

## 5. Conclusions

VSP and/or INO support and high BMI are two independent risk factors for the development of AKI in the early post-cardiac arrest period. The presence of concomitant COVID-19 may also, secondarily, increase the risk of AKI development. The rate of AKI development after cardiac arrest is high, but we did not find that this alone had a direct effect on mortality. The need for VSP and INO is also an independent risk factor for mortality. Although the development of AKI in post-CPR patients is not an independent risk factor for mortality in our study, it may contribute to mortality, prolong hospitalization, and increase healthcare costs. We believe that the sepsis-like picture characterized by intravascular volume depletion, vasodilation, endothelial damage, and microcirculatory disorders after cardiac arrest is the main cause of AKI development and mortality.

## Figures and Tables

**Table 1:** Characteristics of cardiac arrest.

	AKI (*n*: 40)	non-AKI (*n*: 42)	*p*
**Cardiac arrest place (in-hospital)**, *n* (%)	32 (80.0)	30 (71.4)	0.518
**ICU transfer place (emergency depart)**, *n* (%)	26 (65.0)	26 (61.9)	0.951
**Cardiac arrest etiology**, *n* (%)			
Acute coronary syndrome	20 (50.0)	20 (47.6)	1.000
Respiratory (pneumonia, sepsis)	15 (37.5)	12 (28.6)	0.947
Neurological	3 (7.5)	4 (9.5)	
Heart failure	1 (2.5)	3 (7.1)	
Toxemia	0 (0)	2 (4.8)	
Pulmonary embolism	1 (2.5)	1 (2.4)	
**COVID period**, *n* (%)	24 (60)	15 (35.7)	0.048 *
**CPR time** (minute), median (IQR)	15 (10–22)	15 (10–28)	0.877

Mann–Whitney U test; median (IQR); Chi-squared test; *n* (%). * *p* < 0.05.

**Table 2:** Demographic, clinical, and laboratory data of the patients.

	AKI (*n*: 40)	non-AKI (*n*: 42)	*p*
**Age**, median (IQR)	60 (52–71)	60 (42–70)	0.623
**Gender (male)**, *n* (%)	24 (60.0)	29 (69.0)	0.532
**Body mass index**, median (IQR)	27.4 (24.7–34.0)	24.2 (22.9–26.7)	0.001 *
**Charlson comorbidity index**, median (IQR)	3.0 (2.0–5.0)	3.5 (2.0–5.0)	0.948
**Heart rate** (first 1 day average), median (IQR)	85 (74–106)	80 (70–96)	0.199
**MAP** (first 1 day average), median (IQR)	78 (70–84)	81 (71–88)	0.285
**Admission APACHE-II score**, median (IQR)	28 (19–32)	21 (17–26)	0.003 *
**Admission PaO_2_/FiO_2_ ratio**, median (IQR)	163 (108–300)	206 (141–352)	0.095
**Neurological evaluation** (3–4. day), *n* (%)			
GCS (motor < 4),	22 (55.0)	10 (23.8)	0.008 *
Pupillary reflex negativity	5 (12.5)	4 (9.5)	0.735
**Serum creatinine**, median (IQR)			
Basal level	1.1 (0.8–1.3)	1.0 (0.7–1.3)	0.450
After CPR (2 day after)	1.8 (1.2–3.0)	0.8 (0.6–1.0)	<0.001
**ICU Admission lab parameters**, median (IQR)			
pH	7.18 (7.11–7.30)	7.29 (7.16–7.36)	0.015 *
pCO_2_ (mmHg)	50 (39–66)	45 (38–51)	0.177
HCO_3_^−^ (mmol/L)	17.5 (13.0–20.0)	19.0 (16.0–23.0)	0.042 *
Lactate (mmol/L)	4.3 (3.2–6.7)	3.3 (1.9–4.8)	0.021 *
Phosphorus (mmol/L)	5.3 (4.0–7.3)	4.0 (3.0–5.3)	0.005 *
Na (mmol/L)	137 (134–139)	137 (134–140)	0.696
K (mmol/L)	4.3 (3.4–4.6)	4.0 (3.7–4.5)	0.967
CI (mmol/L)	102 (98–108)	102 (99–107)	0.967
AST (U/L)	197 (65–482)	127 (54–240)	0.180
ALT (U/L)	89 (34–243)	74 (41–163)	0.728
WBC (×109/L)	20.6 (13.3–27.4)	15.5 (13.0–24.4)	0.344
Hemoglobin (g/dL)	13.9 (10.9–14.8)	12.8 (11.0–15.1)	0.893
Platelet (×109/L)	283 (192–364)	262 (226–354)	0.735
CRP (mg/L)	11.4 (3.0–67.8)	8.9 (1.5–76.6)	0.578
Glucose (mg/dL)	250 (184–389)	277 (168–321)	0.384
Creatine kinase (max within 48 h)	708 (204–3104)	495 (102–1892)	0.168

Mann–Whitney U test; median (IQR); Chi-squared test; *n* (%): HR: heart rate MAP: mean arterial pressure; APACHE-II: acute physiology and chronic health evaluation; ICU: intensive care unit. * *p* < 0.05.

**Table 3:** Treatment and outcomes of patients.

	AKI (*n*: 40)	non-AKI (*n*: 52)	*p*
**After 24 h fluid status** (Liter), median (IQR)			
Input	3.0 (1.7–4.4)	3.0 (2.5–4.0)	0.564
Output	1.0 (0.4–2.8)	1.9 (1.4–2.7)	0.001 *
Balance	1.5 (0.9–3.1)	0.9 (0.0–2.1)	0.033 *
**First 24 h VSP and/or INO need**, *n* (%)			
No VSP/INO	1 (2.5)	13 (31.0)	<0.001 *
VSP (*n*: 44) or INO (*n*: 4)	24 (60.0)	24 (57.1)	
VSP and INO	15 (37.5)	5 (11.9)	
**First 24 h max body temperature**, median (IQR)	37.5 (37–38.4)	37.4 (36.5–38.0)	0.314
**TTM therapy**, *n* (%)	4 (10.0)	9 (21.4)	0.265
**Continuous RRT in-ICU**	7 (17.5)	5 (11.9)	0.686
**ICU LOS** (day), median (IQR)	5.6 (3.2–9.7)	9.3 (5.7–16.0)	0.020 *
**In ICU mortality**, *n* (%)	26 (65.0)	11 (26.2)	0.001 *

Mann–Whitney U test; median (IQR); chi-squared test; *n* (%). TTM: targeted temperature management; RRT: renal replacement treatment; LOS: length of stay. * *p* < 0.05.

**Table 4:** Reasons that increase the risk of developing AKI after cardiac arrest.

	OR (95%CI)		OR (95%CI)	
	Univariable Analysis	*p*	Multivariable Analysis	*p*
**Age**	1.015 (0.987–1.043)	0.297		
**Male gender**	0.672 (0.271–1.671)	0.393		
**BMI**	1.153 (1.038–1.280)	0.008 *	1.272 (1.089–1486)	0.002 *
**CCI**	1.037 (0.835–1.289)	0.742		
**CPR time**	1.001 (0.966–1.038)	0.946		
**Etiology**				
The others (*n*: 42)	Reference category			
ACS (*n*: 40)	1.100 (0.462–2.616)	0.829		
**COVID-19 period**	2.700 (1.105–6.599)	0.029 *	2.801 (0.859–9.126)	0.088
**Cardiac arrest place**				
In-Hospital (*n*: 62)	Reference category			
Out-of-Hospital (*n*: 20)	0.625 (0.224–1.740)	0.368		
**HR** (first 1 day average)	1.014 (0.992–1037)	0.210		
**MAP** (first 1 day average)	0.977 (0.934–1021)	0.291		
**VSP and INO need**				
No VSP or INO (*n*: 14)	Reference category		Reference category	
VSP or INO (*n*: 48)	13.000 (1.574–107.354)	0.017 *	14.225 (1.172–172.669)	0.037 *
VSP and INO (*n*: 20)	39.000 (4.022–378.199)	0.002 *	42.089 (2.683–660.201)	0.008 *
**TTM therapy**				
Yes (*n*: 13)	Reference category			
No (*n*: 79)	2.455 (0.690–8.731)	0.165		
**Admission Lab Parameters**				
Phosphorus	1.367 (1.086–1.719)	0.008 *	1.237 (0.951–1.609)	0.113
Creatinine	0.884 (0.251–2.223)	0.793		
pH	0.021 (0.001–0.641)	0.027 *		
Lactate	1.263 (1.045–1.526)	0.016 *	1.161 (0.910–1.481)	0.230
WBC	1.023 (0.978–1070)	0.315		
Hemoglobin	1.006 (0.857–1.181)	0.942		
PaO_2_/FiO_2_ ratio	0.998 (0.994–1.001)	0.223		
Glucose	1.001 (0.998–1.005)	0.416		
**Fluid Input**	0.993 (0.806–1.224)	0.950		

CCI: Charlson comorbidity index; ACS: acute coronary syndrome; HR: heart rate; VSP: vasopressor; INO: inotrope; HR: heart rate; MAP: mean arterial pressure. Binary logistics regression analysis, omnibus tests of model: *p* < 0.001; Nagelkerke R-square: 0.524; Hosmer and Lemeshow test: 0.328; accuracy rate: 74.4%, * *p* < 0.05.

**Table 5:** Reasons that increase the risk of developing mortality after cardiac arrest.

	OR (95%CI)		OR (95%CI)	
	Univariable Analysis	*p*	Multivariable Analysis	*p*
**Age**	0.989 (0.963–1.016)	0.414		
**Male Gender** (*n*: 53)	0.662 (0.266–1.646)	0.375		
**CCI**	0.819 (0.651–1.030)	0.087		
**BMI**	1.043 (0.983–1.107)	0.160		
**CPR time**	0.992 (0.957–1.029)	0.681		
**Cardiac arrest causes**				
Non-ACS (*n*: 42)	Reference category			
ACS (*n*: 40)	0.545 (0.226–1.317)	0.178		
**Cardiac arrest place**				
In-Hospital (*n*: 62)	Reference category			
Out-of-Hospital (*n*: 20)	1.741 (0.612–4.951)	0.298		
**HR** (first 1 day average)	1.032 (1007–1058)	0.010*		
**MAP** (first 1 day average)	0.979 (0.937–1023)	0.351		
**VSP and INO need**				
No VSP or INO	Reference category			
VSP or INO	4.667 (0.940–23.158)	0.059	2.863 (0.497–16.495)	0.239
VSP and INO	14.000 (2.370–82.717)	0.004 *	8.455 (1.132–63,160)	0.037 *
**AKI 1–3**	5.234 (2.032–13.482)	0.001 *	2.194 (0.700–6.872)	0.178
**GCS motor response < 4**	5.133 (1.962–13.428)	0.001 *	5.004 (1.590–15.750)	0.006 *
**Pupillary reflex negativity**	12.138 (1.441–102.247)	0.022 *		
**No TTM therapy**	3.238 (0.820–12.792)	0.094		
**Admission Lab Parameters**				
Lactate	1.239 (1.032–1.488)	0.022 *		
PaO_2_/FiO_2_ ratio	0.995 (0.992–0.999)	0.023 *	0.995 (0.990–0.999)	0.025 *
WBC	1.005 (0.962–1.049)	0.838		
Hemoglobin	0.854 (0.721–1.011)	0.066		
Glucose	1.000 (0.996–1.003)	0.982		
**Fluid Input**	1.241 (0.965–1.594)	0.092		
**CRRT therapy**	2.828 (0.778–10.282)	0.115		

CCI: Charlson comorbidity index; ACS: acute coronary syndrome; VSP: vasopressor; INO: inotrope; HR/MAP: heart rate/mean arterial pressure; CRRT: continuous renal replacement therapy. Binary logistics regression analysis, omnibus tests of model: *p* < 0.001; Nagelkerke R-square: 0.411; Hosmer and Lemeshow test: 0.510; accuracy rate: 80.5%, * *p* < 0.05.

## Data Availability

The data that support the findings of this study are available from the corresponding author upon reasonable request.
